# The People’s Journal

**Published:** 1863-10

**Authors:** 


### THE PEOPLE'S JOURNAL.

The fourth number of this deservedly popular Journal is at hand, and it is certainly not inferior to its predecessors either in style or matter. The articles are brief and to the point, and upon subjects, for the discussion of which there is the most pressing necessity. The people must be informed upon all points connected with the welfare of their teeth, and there is no better way of doing it, than through a medium of this kind. We have heard the "People's Dental Journal" well spoken of everywhere. Our patients like it very much, and *take to it naturally*.

